# Generative artificial intelligence use and interpersonal educational interaction among nursing students: self-regulated learning as a statistical mediator

**DOI:** 10.3389/fpsyg.2026.1864535

**Published:** 2026-10-20

**Authors:** Jianyun Li, Ershan Xu, Li Li, Lijuan Huang, Yan Pu, Ruirui Huang

**Affiliations:** 1 Faculty of Nursing, Guangxi University of Chinese Medicine, Nanning, China; 2 School of Nursing, Hunan Medical College, Huaihua, China

**Keywords:** educational interaction, generative artificial intelligence interaction, mediation analysis, nursing students, self-regulated learning

## Abstract

**Background:**

Generative artificial intelligence (GAI) is reshaping health sciences education. For nursing students, educational interaction (EI), self-regulated learning (SRL), and GAI interaction are all particularly important. However, these relationships and their processes remain underexplored.

**Methods:**

A total of 1020 nursing students were recruited from one medical university in Hunan Province in China. The Demographics Questionnaire, the Chinese version of Student-GAI interaction scale, the Educational Interaction Scale, and the Self-regulated Learning Questionnaire were administered. SPSS 22.0 and AMOS 24.0 software were used to analyze the data.

**Results:**

The mean scores of student-GAI interaction (SGI) and EI were 14.80 ± 2.27 and 70.90 ± 8.15, respectively. Significant positive relationships were found among all three variables (all *P* < 0.01). Both SGI (β = 0.256) and SRL (β = 0.520) positively were associated with EI (both *P* < 0.001). Furthermore, SGI showed a significant direct association with EI, with a direct effect value of 0.92 (*P* < 0.001). SRL statistically mediated the association between SGI and EI, with a significant indirect effect value of 0.44. The mediating effect represented 32.35% of the total effect (*P* < 0.001). Dimension-specific analyses revealed that the mediating effect was strongest for student-teacher interaction (45.41%) and student-content interaction (41.54%).

**Conclusions:**

SGI is positively associated with EI among nursing students, both directly and indirectly through SRL. Educational strategies that encourage effective GAI use and strengthen SRL may be associated with enhanced EI in nursing education.

## 1 Introduction

The foundation of generative artificial intelligence (GAI), laid in the 1940s, achieved groundbreaking advancements by 2022, leading to the development of services like ChatGPT and DeepSeek (Babu et al., 2024; Gibney, 2025; Stokel-Walker, 2023). While these GAI systems solve problems and offer structured responses (Gibney, 2025; Lim et al., 2023). Their integration into education has sparked debates about the role of traditional human connection and raised discussions about the nature of human-AI vs. human-human interaction in educational settings (Else, 2023; Stokel-Walker, 2023).

In education, GAI tools have demonstrated their unprecedented ability in natural language understanding, content generation, and adaptive feedback across many disciplines (Preiksaitis and Rose, 2023). GAI's educational applications extend earlier technology integration paradigms, including Clark's media effects principle (Clark, 1983), Jonassen's constructivist learning tools framework (Jonassen, 1995), and Laurillard's conversational model (Laurillard, 2002). Unlike traditional intelligent tutoring systems that merely deliver pre-programmed content, GAI personalizes instruction while dynamically generating novel educational materials. It is widely used in nursing fields, especially in nursing education (Clark, 1983). For example, GAI offers potential benefits such as assisting students with literature review, drafting, and revision of assignments, providing realistic patient scenarios for practice, and enabling personalized, interactive learning experiences (Preiksaitis and Rose, 2023; Topaz et al., 2024). However, GAI also poses challenges to education. Several scholars have voiced concerns that GAI may potentially disrupt students' interpersonal interactions (Zhou and Zhao, 2024) and lead to other adverse effects, including over-reliance on AI-generated content, reduced critical thinking, academic dishonesty, and diminished face-to-face communication skills (Ryznar, 2023). Hence, how this new interaction impacts students remains a topic that requires further discussion.

To understand how GAI specifically impacts nursing students, it is necessary to distinguish nursing-specific GAI applications from generic higher-education use. While GAI tools have been widely discussed in general higher education, their application in nursing education presents distinctive characteristics. A recent topic modeling analysis reveals that nursing GAI research clusters around simulation education, assignment support, exam performance, and patient education, with particular emphasis on clinical judgment and patient safety reasoning through AI-generated scenarios (Seo and Kim, 2024). However, this literature predominantly frames GAI as an opportunity, with limited attention to its risks and limitations, suggesting that nursing-specific GAI use extends beyond generic higher-education technology adoption patterns and warrants distinct examination.

This distinct examination requires a theoretically grounded understanding of how GAI shapes student learning processes. Research has increasingly framed SRL (SRL) as a cyclical process in which GAI interaction may differentially shape specific phases and dimensions. Zimmerman's (2000) model identifies three phases expected to be influenced: forethought (goal setting and strategic planning), performance (task execution and metacognitive monitoring), and self-reflection (self-evaluation and adaptation). While GAI can scaffold this full cycle and foster learner autonomy, defined as the capacity to take charge of one's own learning with decreasing external support (Little, 1991), this scaffolding may create a performance-competence gap. A recent meta-analysis indicates that GAI's capacity to support immediate task performance may outpace its ability to cultivate durable, transferable self-regulatory competence (Achuthan, 2025). This raises the question of whether GAI fosters genuine autonomy or merely optimizes performance execution, a distinction critical for educational contexts where independent self-regulation is the ultimate goal.

## 2 Background

Interaction is a complex concept and has been deemed as one of the important ingredients in all forms of education, regardless of whether technology is involved (Edirne and Ozturk, 2024). Wagner defined interactions as a mutual exchange that involves at least two entities and two corresponding actions (Wagner, 1994). Moore identified the major constituents of interaction in education as learner-instructor interaction, learner-learner interaction, and learner-content interaction (Moore, 1989). In this article, educational interaction (EI) encompasses Moore's three types of interaction, where the term “learner” refers to students. The concept of interaction has been expanded to nursing education, where various technologies facilitate educational exchanges by serving as intermediaries between learners, instructors, and content (Cho and Kim, 2017; Salehian et al., 2017; Summers et al., 2024). While the EIs among nursing students typically involve student-instructor, student-student, and student-content dynamics, the rapid development of GAI has introduced the concept of student-GAI interaction (SGI; Summers et al., 2024).

SGI refers to the dynamic exchange and engagement between students and GAI systems in educational contexts (Liang et al., 2023). This interaction encompasses how students use, communicate with, and learn from GAI tools like ChatGPT, DeepSeek, or other generative models to achieve educational goals. It includes activities such as asking questions, receiving feedback, collaborating on tasks, and utilizing for problem-solving, content creation, or personalized learning (Holmes et al., 2019; Lodge et al., 2023; Luckin, 2017; Wagner, 1994). Garavaglia's editorial (Garavaglia, 2024) synthesizes some effects of GAI on teacher-student interaction, including role transformation (educators' shift from delivering content to curating AI-generated materials), enhanced interactivity (through feedback loops and personalized scaffolding), and emerging challenges (e.g., academic integrity risks). Moy and Feldstein claimed that the rapid advancements in GAI are transforming how students and faculty engage with content and interact with one another (Moy and Feldstein, 2024). SGI may be one of the significant factors influencing EI.

SGI and student-instructor interaction are closely related. Student-instructor interaction refers to a two-way communication between the instructor of the course and learners (Garavaglia, 2024). This type of interaction is central to the teaching and learning process and is critical for achieving educational goals (Moy and Feldstein, 2024). It has a profound impact on students' satisfaction with learning and their academic achievements (Seo et al., 2021). It is featured in communication, feedback, and support (Moy and Feldstein, 2024). Kyoungwon Seo and colleagues interviewed and analyzed responses from 12 students and 11 instructors to gather their genuine perspectives on various applications of Artificial Intelligence (AI) systems in online learning environments. The findings indicate that participants view AI systems as capable of facilitating large-scale, personalized interactions between learners and instructors. These systems have received positive evaluations for their ability to enhance the quality of communication, provide timely and personalized support in extensive educational settings, and strengthen the sense of connection (Seo et al., 2021). Recent studies have yielded mixed findings: while some report that AI systems facilitate personalized instructor feedback at scale (Alowais et al., 2023; Dushyanthen et al., 2025), others caution that over-reliance on AI-mediated communication may reduce the depth of human connection in teaching (Choi, 2026).

There is also a relationship between student-student interaction and SGI (Tang, 2024). Student-student interaction refers to collaboration on learning tasks, where students work together, share knowledge, and help each other understand course material (Scriven, 1996). GAI can be integrated into collaborative learning environments, not only supporting the individual learner but also enhancing interactions between learners. GAI systems can facilitate group discussions, encourage participation, and offer personalized feedback during group activities, thus promoting student-student interaction (Wardat et al., 2024).

According to Moore, student-content interaction refers to a one-way process in which learners engage with the subject matter or course content through elaboration and reflection. Students have to construct their knowledge through a process of accommodating new information into previously existing cognitive structures (Moore, 1989). The relationship between SGI and student-content interaction is evolving rapidly (Chen and Anyanwu, 2025). GAI enhances student-content interaction by personalizing and adapting content based on learner needs and performance (Skjuve et al., 2021). Through dynamic feedback, real-time content generation, and customized learning paths, GAI systems create more engaging and effective learning experiences (Li et al., 2024). GAI tools bridge static content and personalized learning, enhancing cognitive engagement and learning outcomes.

Some researchers held a positive attitude toward the use of GAI (Garavaglia, 2024; Moy and Feldstein, 2024). However, some educators claimed that the strong human-computer interaction capabilities of GAI may dissolve the deep dialogue and emotional resonance traditionally found between teachers and students, as well as among students themselves, and potentially weakening social-emotional skills (Zhou and Zhao, 2024). Based on the above discussion, we hypothesized that:

Hypothesis 1: SGI positively predicts EI among nursing students.

Although existing research has identified preliminary connections between SGI and EI, there remains a need for more empirical studies to examine the specific impacts of GAI integration on EI, and the mediating factors underlying these connections. Studies have implicated that SRL is related to EI and SGI, separately. The relationship between self-regulation learning and SGI is crucial in modern educational settings. SRL is originally from psychology and was defined by Bandura in terms of three forms of cognitive motivators including causal attributions, outcome expectancies, and cognized goals which refer to mental representations of desired future outcomes that individuals consciously adopt to guide and sustain their behavior (Bandura, 1988). Cognized goals Researchers in education-related fields have gradually adopted the concept of self-regulation from psychology and adapted it to student learning and educational practice, thereby forming the concept of self-regulated learning, which concerns the motivation and learning strategies that students employ to achieve their learning goals (Schunk, 2005). GAI can significantly enhance SRL by providing personalized learning paths (Wei et al., 2024), and real-time feedback (Lai, 2024). GAI helps students manage their cognitive processes, monitor their progress, and adapt their strategies, thereby fostering greater autonomy and more effective learning (Halkiopoulos and Gkintoni, 2024). Based on the above discussion, we hypothesized that:

Hypothesis 2: SGI positively predicts SRL.

The relationship between SRL and EI is well-documented (Sankalaite et al., 2021). Students who are more self-regulated require less external motivation from EI (Massa et al., 2005). Teacher-student and peer interactions offer opportunities for learners to reflect on their learning, adjust strategies, and gain motivation, all of which contribute to enhanced SRL (Torres et al., 2024). Based on the above discussion, we hypothesized that:

Hypothesis 3: SRL positively predicts EI.

This study integrates Information Processing Theory (IPT) with Social Cognitive Theory (SCT) to explain how SGI affects EIs via SRL (Bandura, 2001; Miller, 1956). IPT was developed by American psychologists including George Miller in the 1950s. IPT explains the information processing including the sensory register, short-term memory, working memory active transformation of encoded information and long-term memory (Swanson, 1987). It elucidates the cognitive mechanisms: GAI optimizes attention through adaptive content presentation, supports working memory via chunking and multimodal representations, and strengthens long-term retention through spaced repetition and contextualized practice - collectively improving information processing efficiency (Swanson, 1987).

SCT, initially proposed by Bandura, complements this by explaining motivational pathways, where GAI interactions build self-regulation skills that promote active engagement (Bandura, 2001). SCT demonstrates that human behavior is explained through the interplay of three factors: personal factors, behavioral patterns, and environmental influences. Within SCT, SRL mediates the relationship between SGI and educational outcomes through triadic reciprocal causation: personal factors, behavioral factors, and environmental factors. In the context of nursing students, SRL is part of the personal factors. The way students interact with GAI or traditional methods reflects their behavioral patterns. The learning environment, including the availability of GAI tools and traditional teaching methods, shapes how students interact and learn.

Together, these theories reveal a dual-pathway effect: GAI directly improves cognitive processing (IPT pathway) while indirectly fostering better EI through enhanced SRL (SCT pathway). This integration is particularly relevant for nursing education, where GAI's ability to simulate clinical scenarios simultaneously trains cognitive skills and emotional regulation, demonstrating how technological, cognitive, and behavioral factors interact to shape learning outcomes. Based on this, we hypothesized that:

Hypothesis 4: SRL mediates the relationship between SGI and EI.

Hence, this study aims to (1) examine the relationships among SGI, EI, and SRL, and (2) identify the statistical mediating effect of SRL on the relationship between SGI and EI among nursing students.

## 3 Materials and methods

### 3.1 Study design and participants

A cross-sectional survey was conducted. The participants of this study were a sample of nursing students from the Hunan Medical College in Hunan Province, recruited from January 6th to January 16th, 2025. Convenience sampling was employed to select the participants. The inclusion criteria were as follows: (1) undergraduate students majoring in nursing; (2) willingness to participate in the study. The exclusion criteria were: (1) students on leave of absence from school; (2) fourth-year nursing interns who were not on campus.

The researchers designed the electronic survey questionnaire using the Questionnaire Star web platform and distributed the questionnaire links to the class leaders of nursing students who had undergone questionnaire collection training. The class leaders then forwarded the survey links to their respective class WeChat groups. All participants voluntarily clicked on the online survey link and were informed about the research's purpose, significance, data security, and their rights and responsibilities. They were also required to truthfully report their interactions with GAI, EI, and SRL experiences over the past semester. Each participant was only able to fill out the questionnaire once, and any questions encountered during the online filling process were answered by members of the research team. The collected data were strictly confidential and used solely for research purposes. This study was approved by the Medical Ethics Committee of the Hunan Medical College (Approval No.: 2025/H01001).

The study included 9 independent variables, and the sample size was set to be 20 times the number of independent variables (Zhenqiu and Yongyong, 2014). Considering a potential sample loss rate of 20%, a minimum sample size of 216 was required. A total of 1573 nursing students completed the questionnaire online. However, 553 questionnaires were excluded from data analysis because the respondents' completion time was less than 100 s, their responses exhibited zero standard deviation across all items (row_sd = 0) indicating invariant responding, or displayed cyclic response patterns, all of which were deemed indicative of questionable response quality. Ultimately, 1,020 valid questionnaires were analyzed.

### 3.2 Measurement

#### 3.2.1 Demographics questionnaire

This questionnaire includes items related to the demographic and professional characteristics of participants, including age, gender, grade, and the earliest time they began using GAI for learning.

#### 3.2.2 Student–GAI interaction scale

The Student – GAI Interaction Scale, created by Jing Liang and colleagues in 2023, assesses how students interact with GAI in their learning activities (Liang et al., 2023). The scale comprises four items: (1) “Student–GAI interaction is becoming more frequent in my course learning”; (2) “When I have questions during course learning, I use GAI to seek answers”; (3) “I use GAI to ask for advice when completing course tasks”; (4) “My classmates are becoming more interested in using GAI tools.” Responses are assessed using a 5-point Likert scale, where 1 indicates “strongly disagree” and 5 indicates “strongly agree.” Participants with no experience interacting with GAI can mark the question as 1. Higher scores suggest more interaction between students and GAI, while lower scores indicate less experience with GAI during learning. The scale was subjected to rigorous scrutiny by a panel of 17 researchers, experts, and educators in the relevant field. Based on their detailed feedback regarding questions that were deemed complex, unclear, or ambiguous, the scale was refined to enhance its content validity (Liang et al., 2023). The scale was validated among 389 undergraduates and has a Cronbach's alpha value of 0.974 (Liang et al., 2023). In this study, it achieved a Cronbach's alpha of 0.780. The confirmatory factor analysis (CFA)showed CMIN/DF = 5.422 (*df* = 2), reflecting the near-saturated nature of the 4-item unidimensional model. Model fit was therefore primarily evaluated using robust indices: RMSEA = 0.066, SRMR = 0.0172, IFI = 0.992, TLI = 0.975, CFI = 0.992, and NFI = 0.990. Standardized factor loadings ranged from 0.673 to 0.705 (all *p* < 0.001). Composite reliability (CR) was 0.783 and average variance extracted (AVE) was 0.474.

#### 3.2.3 Educational interaction scale

The Educational Interaction Scale was used to measure the extent of interactions among students, between students and teachers, and between students and content in learning environments. The original scale was developed by Kuo, Walker, and colleagues (Kuo et al., 2014). This scale comprises 18 items across three factors: student-student interaction with eight items, student-teacher interaction with six items, and student-content interaction with four items. It employs a 5-point Likert scale, where 1 signifies “completely disagree” and 5 signifies “completely agree.” Higher scores indicate greater perceived interaction. The total scale's Cronbach's alpha was 0.908 (Kuo et al., 2014). In our study, the scale achieved a Cronbach's alpha of 0.925. Confirmatory factor analysis indicated a suboptimal model fit: χ^2^/df = 10.902, RMSEA = 0.099, SRMR = 0.056, CFI = 0.872, and TLI = 0.852. Standardized factor loadings for the 18 items ranged from 0.547 to 0.848 (all *p* < 0.001). The CR was 0.887, 0.860, and 0.862 for student-student, student-teacher, and student-content interaction, respectively; the AVE was 0.496, 0.511, and 0.611.

#### 3.2.4 Self-regulated learning questionnaire

The questionnaire used in this study was revised by Zhang Chunmei in 2007 (Zhang, 2007). The original scale was developed by Schunk and Zimmerman in 1999 (Schunk and Ertmer, 1999). It was used to measure the extent of the self-regulated learning. The revised version consists of 15 items and employs a 5-point Likert scale, where 1 indicates “strongly disagree” and 5 indicates “strongly agree.” Higher scores reflect stronger SRL abilities. In the present study, this questionnaire obtained a Cronbach's alpha of 0.919. Confirmatory factor analysis yielded: χ^2^/*df* = 9.221, RMSEA = 0.090, CFI = 0.896, TLI = 0.879, and SRMR = 0.050. Standardized factor loadings ranged from 0.544 to 0.777 (all *p* < 0.001). The CR was 0.921 and the AVE was 0.440.

### 3.3 Data analysis

The data were analyzed using SPSS 22.0 and AMOS 24.0. Categorical data, such as the characteristics of participants, were presented as frequencies or percentages. Continuous data, such as the levels of EI, SGI, and SRL, which were assumed to follow a normal distribution, were described using means and standard deviations. Pearson correlation analysis was performed to examine the relationships between EI, SGI, and self-regulated learning. Stepwise regression analysis was used to explore the predictive effects of SGI and SRL on EI. Common method bias was assessed using Harman's single-factor test. The first unrotated factor explained 33.57% of the variance, below the 40% criterion (Podsakoff et al., 2003), indicating that common method bias was unlikely to substantially affect the results.

Structural equation modeling (SEM) was conducted using AMOS 24.0 to perform confirmatory factor analysis for the scales and to construct a mediating effect model of the relationships among EI, SGI, and SRL. The size and significance of the mediation effects were analyzed using the Bootstrap method (Chernick, 2008). Confidence intervals for indirect effects were calculated through Bootstrap repeated sampling to determine the statistical significance of mediating effects. Mediation effects were considered significant if the confidence intervals did not include zero. A *p*-value of less than 0.05 was used as the threshold for statistical significance.

## 4 Results

### 4.1 Characteristics of participants

The age of nursing students ranged from 17 to 24 years, with a mean age of 19.09 ± 2.40 years. The majority were female (*n* = 849, 83.2%), while 16.8% were male (*n* = 171). No participants identified as other genders, as the demographic questionnaire provided only “male” and “female” as response options for gender. Among the 1020 participants, 46.0% (*n* = 469) were first-year students, 32.7% (*n* = 334) were second-year students, and 21.3% (*n* = 217) were third-year students. Additionally, 98.8% of the students reported having begun to use GAI for course learning by 2024.

### 4.2 Descriptive and correlation analysis

Table 1 presents the means, standard deviations, and Pearson correlation coefficients for the main indicators. The results indicate that EI, SGI, and SRL are positively correlated with each other. Statistically significant correlations are observed between EI and SGI (*r* = 0.378, *p* < 0.01), SGI and SRL (*r* = 0.236, *p* < 0.01), and SRL and EI (*r* = 0.580, *p* < 0.01).

**Table 1:** Descriptive statistics and Pearson correlation coefficient (*n* = 1,020).

Variables	Mean ±SD	1	2	3
1.EI	70.90 ± 8.15	1.000		
2.SGI	14.80 ± 2.27	0.378^**^	1.000	
3. SRL	56.00 ± 7.34	0.580^**^	0.236^**^	1.000

^**^p < 0.01.

### 4.3 Regression estimates

Prior to regression analysis, the assumptions of linear regression were systematically examined. The Durbin-Watson statistic was 1.981, indicating independence of residuals. Residual normality was assessed by histograms and P-P plots; visual inspection confirmed approximate normality with minor positive skewness at the right tail. Multicollinearity was absent (VIF = 1.059, tolerance = 0.944). The residual scatterplot supported homoscedasticity and linearity. Maximum Cook's distance was 0.074, well below the conventional threshold of 1, confirming no influential cases; maximum standardized residual was 4.191, and maximum studentized residual was 4.212, but model robustness was unaffected. Centered leverage value reached 0.039, with no problematic high-leverage observations.

Using the total score of EI as the dependent variable, and the scores of SGI and SRL as predictor variables, a stepwise regression analysis was conducted. The results showed that the SGI and SRL were included in the regression equation. The regression model had a coefficient of determination *R*^2^ = 0.398, with an adjusted coefficient of determination Δ*R*^2^ = 0.397. The results indicated that both the SGI and SRL were significant predictors of EI and were included as independent variables in the regression model. The SGI and SRL were positively associated with EI, as shown in Table 2.

**Table 2:** Linear regression analysis for the EI (*n* = 1,020).

Covariate	B	SE	*B*	*t*	*p*	VIF
SGI	0.919	0.090	0.256	10.224	< 0.001	1.059
SRL	0.577	0.028	0.520	20.758	< 0.001	1.059

B, unstandardized regression coefficient; SE, standard error; β, standardized regression coefficient.

### 4.4 Analysis of mediating effect

Based on the results of correlation and regression analyses, two mediating effect models were conducted. Model 1 (Figure 1) examined the mediating effect of SRLon the relationship between SGI and the total score of EI. SGI positively predicted SRL (β = 0.24), which in turn positively predicted EI (β = 0.52). The direct effect of SGI on EI was also significant (β = 0.26). Thus, two pathways are associated with EI: Path 1 (standardized direct effect: β = 0.26) and Path 2 (standardized indirect effect:β = 0.12). These findings support Hypotheses 2 and 3.

**Figure 1:**
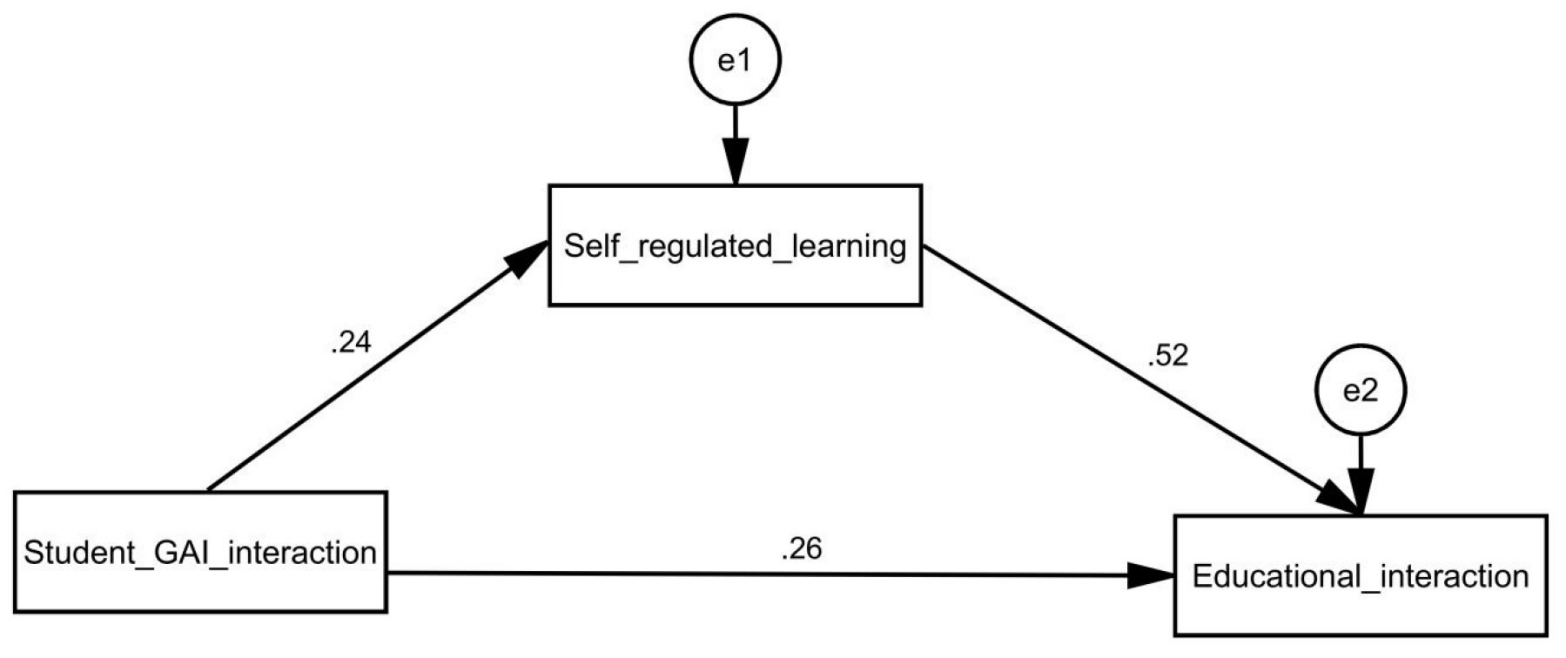
The mediating role of SRL in the relationship between SGI and EI. Standardized estimates (β) are shown. The indirect effect of SGI on EI via SRL is 0.12, accounting for 32.35% of the total effect.

Model 2 (Figure 2) extended this to three dimensions of EI. The standardized direct effects of SGI on the three dimensions were also significant (β = 0.32, 0.14, and 0.16, respectively). The standardized indirect effects of SGI on the three dimensions of EI via SRL were 0.09, 0.12, and 0.15.

**Figure 2:**
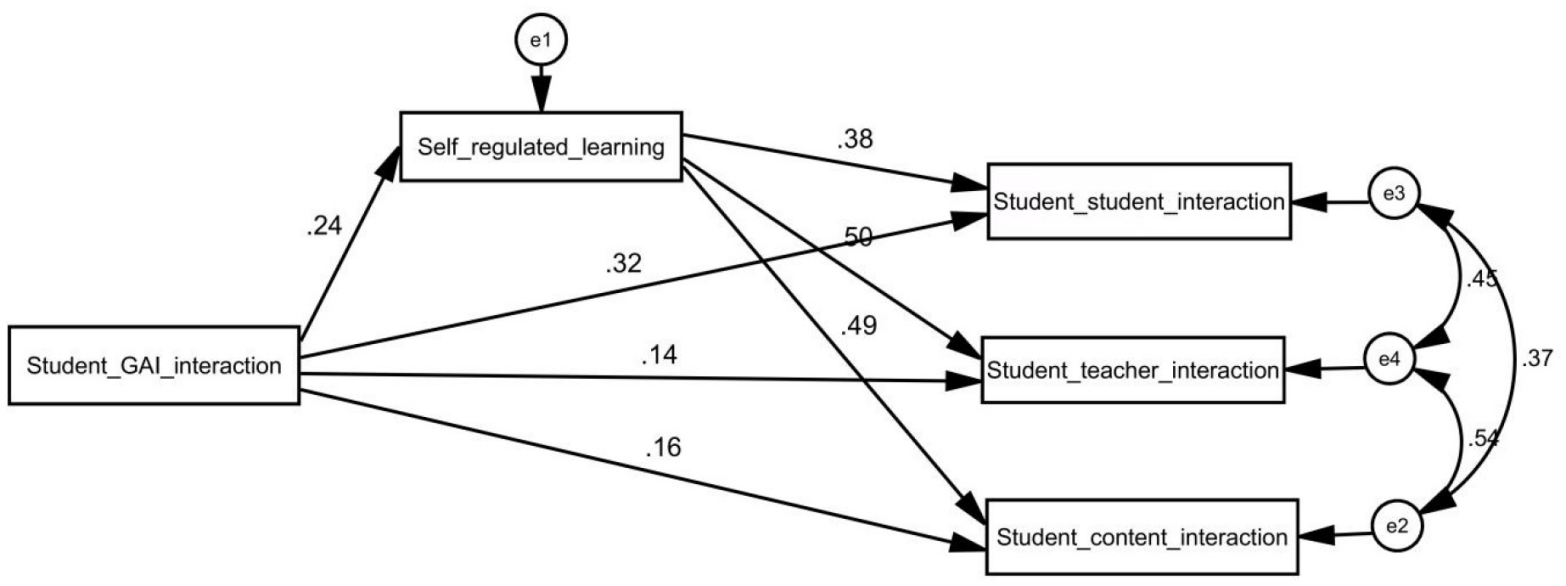
The mediating role of SRL in the relationship between SGI and three dimensions of EI. Standardized estimates (β) are shown. The indirect effects of SGI on the three dimensions of EI via SRL are 0.09, 0.12, and 0.15 (22.15%, 45.41%, and 41.54% of the total effect, respectively).

As both models were just-identified, with χ^2^ values of zero, GFI and CFI both equal to 1.00, and RMSEA being meaningless, conventional fit indices were not applicable.

The mediating effect was statistically tested using the Bias-Corrected Bootstrap method, as shown in Table 3. Results of mediation analysis using the 5,000 bootstrap sampling method showed that the total effect of SGI on EI was 1.36 (95% CI [1.14, 1.57], *p* < 0.001). This total effect can be decomposed into direct and indirect components. Specifically, the direct effect of SGI on EI was 0.92 (95% CI [0.72, 1.11], *p* < 0.001). The indirect effect of SGI on EI through SRL was 0.44 (95% CI [0.32, 0.57], *p* < 0.001). The confidence interval for the indirect effect of SGI on EI did not include 0, indicating that the mediating effect of SRL between SGI and EI was statistically significant. This supports Hypothesis 4. Meanwhile, the confidence interval for the direct effect of SGI on EI also did not include 0, suggesting that SGI significantly and positively associated with EI. This supports Hypothesis 1 and also suggests that SRL functions as a partial mediator. The indirect effect represented 32.35% of the total effect, while the direct effect accounted for 67.65% of the total effect.

**Table 3:** Bootstrap results for the mediation effect (*n* = 1,020).

Outcome	Mediating path	Effect (unstandardized)	SE	*B* (standardized)	p	LLCI	ULCI	Relative effect size (%)
Total score	Indirect effect	0.44	0.06	0.12	0.001	0.32	0.57	32.35
	Direct effect	0.92	0.10	0.26	0.001	0.72	1.11	67.65
	Total effect	1.36	0.11	0.38	0.001	1.14	1.57	
Student-student interaction	Indirect effect	0.16	0.03	0.09	0.001	0.11	0.21	22.15
	Direct effect	0.56	0.06	0.32	0.001	0.45	0.68	77.85
	Total Effect	0.72	0.06	0.41	0.001	0.60	0.84	
Student-teacher interaction	Indirect effect	0.17	0.02	0.12	0.001	0.13	0.22	45.41
	Direct effect	0.21	0.04	0.14	0.001	0.13	0.29	54.59
	Total effect	0.38	0.04	0.26	0.001	0.29	0.47	
Student-content interaction	Indirect effect	0.11	0.02	0.15	0.001	0.08	0.14	41.54
	Direct effect	0.15	0.03	0.16	0.001	0.10	0.20	58.46
	Total effect	0.26	0.03	0.28	0.001	0.20	0.32	

Effect, unstandardized estimate; β, standardized estimate; Bootstrap confidence intervals are based on unstandardized estimates.

Dimension-specific analyses revealed that the mediating effect of SRL was strongest for student-teacher interaction (45.41%) and student-content interaction (41.54%), but weakest for student-student interaction (22.15%). This suggests that was GAI use primarily associated with EI via teacher-related and content-related channels, with SRL playing a more limited role in peer interaction.

## 5 Discussion

### 5.1 Strengths of the Study

This study makes a timely contribution to the ongoing global debate about integrating GAI into higher education. While some educators and institutions have resisted incorporating GAI tools into the classroom or have pointed to a lack of empirical evidence regarding their potential associations with learning, our findings are consistent with a potential beneficial association of GAI in educational settings. Specifically, we found that SGI was associated with SRL and was linked to EI among nursing students. This evidence is particularly valuable given the ongoing expansion of GAI use in universities worldwide and the pressing need for data-driven guidance on its pedagogical implementation.

This study explores the relationship between nursing students' interaction with GAI and EI, with a particular focus on the mediating role of self-regulated learning. The empirical study collected responses from questionnaires completed by 1020 nursing students. The main findings of this study are as follows: (1) The mean scores of SGI and EI were 14.80 (SD = 2.27) and 70.90 (SD = 8.15), respectively. (2) Overall, there is a significantly positive and direct relationship between the degree of student–GAI interaction and EI. (3) This positive relationship is mediated by SRL, with a significant mediation effect value of 0.44, accounting for 32.35% of the total effect. (4) The mediating effect varies across dimensions: strongest for student-teacher interaction (45.41%) and student-content interaction (41.54%), but weakest for student-student interaction (22.15%).

In this study, the mean score of SGI was 14.80 (SD = 2.27), and 98.8% of students reported beginning to use GAI for course learning by 2024. This suggests that nursing students showed a high level of acceptance toward GAI tools and reported engaging with them during their learning activities. This is similar to the findings on student GAI interaction levels from a 2023 survey of 389 Chinese undergraduates across diverse grades, provinces, schools, and majors (Liang et al., 2023). The high acceptance and active engagement with GAI tools were associated with their unique advantages in nursing education. Firstly, GAI tools can provide personalized learning experiences, which are associated with students‘ understanding of complex nursing knowledge (Wang et al., 2025). Secondly, the perceived usefulness, ease of use, and interactivity of GAI tools were associated with students' active participation (Hawk et al., 2025; Sami et al., 2025). A cross-sectional study found that the majority of 702 Pakistani medical students regarded AI as an effective and credible educational tool in medicine, which was associated with learning, including shorter study time, access to accurate answers, and improved study outcomes (Sami et al., 2025).

This study found that SGI was positively and directly associated with EI among nursing students. The regression analysis results indicated that SGI was positively associated with EI (β = 0.256, *P* < 0.001). The mediation model indicated that SGI showed a direct association of 0.92 with EI, accounting for 67.65%. This positive relationship is consistent with the possibility that GAI use may be associated with the overall quality of EIs in nursing education, empirically supporting a potential beneficial association with students' learning. This aligns with some empirical studies (Hawk et al., 2025; Liang et al., 2023; Shimizu et al., 2023) and opinion articles (Baidoo-Anu and Ansah, 2023; Zhou and Zhao, 2024) that highlight the potential of GAI to contribute to teaching and learning. For instance, it is associated with students' comprehension of the learning material and with their knowledge mastery (Baidoo-Anu and Ansah, 2023), is associated with students' self-efficacy and cognitive engagement (Liang et al., 2023), and is linked to their academic achievement.

SRL is of critical importance in medical education. This study examines the statistical mediation of nursing students' SRL in the relationship between SGI and EI. The mediation model indicated that SRL showed a mediating effect of 0.44, accounting for 32.35% of the total effect. This suggests that SGI was directly associated with EI and also indirectly linked to it through self-regulated learning, thus functioning as a partial statistical mediator. Furthermore, the mediating effect varies across dimensions: strongest for student-teacher interaction (45.41%) and student-content interaction (41.54%), but weakest for student-student interaction (22.15%).

Similarly, Wang Siyao et al.'s recent study results indicated that SRL functioned as a full statistical mediator in the relationship between GAI support and college students' creativity, with a positive effect. Specifically, GAI use was associated with SRL among college students, which in turn was linked to lower negative associations with their creativity (Wang and Huang, 2024). A meta-analysis in 2024, including 61 medical education studies, showed a positive and significant correlation between SRL and overall learning outcomes (i.e., affective, test scores, behavioral outcomes, and mental health outcomes; Zheng and Sun, 2025).

The results indicate significant statistical mediation through self-regulated learning, though the directionality of these associations cannot be definitively established given the cross-sectional design. The effectiveness of SGI may be associated with learning motivation and technology acceptance, which are not measured in this study. Future research should examine how individual differences shape these associations. For instance, students with intrinsic motivation may be better positioned to harness GAI for deep learning, while those with lower technology acceptance might experience these interactions as frustrating rather than empowering.

The finding in this study aligns with the theoretical framework of SCT, which proposes that optimizing personal factors (e.g., self-regulated learning), behavioral patterns (e.g., active utilization of GAI), and environmental factors (e.g., supportive learning environments) may be associated with the quality of EI. However, we acknowledge that GAI adoption in education is a multi-level process extending beyond the student level. Drawing on the ADO framework (Antecedents, Decisions, Outcomes; Raman et al., 2026), our student-level variables (SGI, SRL, EI) represent the outcome level of a broader system. Critical antecedents—including institutional policies, technological infrastructure, teacher training, and curriculum design—are associated with the contexts in which students engage with GAI. Decision-level processes, such as motivational beliefs, cognitive engagement strategies, and trust calibration (Klingbeil et al., 2024), are linked to how these antecedents are associated with student outcomes. Notably, high GAI interaction frequency may reflect adaptive trust, overreliance, convenience-seeking, or course-mandated use—patterns with distinct implications for learning. AI literacy and appropriate reliance were not assessed in this study but represent critical explanatory variables for future research. Our cross-sectional data limit empirical testing to the student level, but our findings indicate that future research should consider multi-level modeling or mixed-methods designs to examine how institutional and technological antecedents are associated with student-level GAI adoption and EI. These results are consistent with the potential use of GAI in nursing education and may inform theoretical considerations for potential educational technologies.

### 5.2 Limitations

Several limitations of this study should be noted. First, this study reports the perspectives of nursing students from a single medical university in China, which may limit the generalizability of the findings to a broader population of nursing students. It is recommended that future research be conducted with larger samples and multiple centers to enhance the generalizability of the findings. Second, this study adopts a cross-sectional survey design. This limits causal inference. Future research could utilize longitudinal designs or experimental designs to meticulously explore causal pathways, thereby providing further validation and deepening these preliminary findings. The propensity score matching or instrumental variable analysis could help address self-selection bias and strengthen causal inference in future studies. Third, the data for this study were collected using scales, primarily relying on self-reports from nursing students, which may introduce potential biases such as social desirability bias. Fourth, this study inadequately controlled for potential confounders. Beyond basic covariates (age, gender, grade, earliest GAI usage time), key factors at three levels were omitted: individual (prior academic achievement, AI literacy, learning motivation, digital access), GAI usage (frequency, purpose, platform type, task complexity, teacher-guided vs. self-initiated use), and institutional (course type, teacher support, permission policies). These omissions may introduce residual confounding; for instance, AI literacy may independently influence GAI interaction patterns, and teacher-guided use may differentially influence EI. Future studies should incorporate these multi-level variables as controls or moderators.

### 5.3 Implications

Based on the findings of this study, how to safely and effectively enhance the interaction between nursing students and GAI in ways that may be associated with traditional EI has emerged as an urgent issue in educational practice. GAI is being integrated into the way we teach and learn (Liang et al., 2023; Liu et al., 2024). Nursing educators should innovate human-computer collaborative teaching methods and consider integrating GAI tools into various teaching components. Notably, the dimension-specific findings suggest that educators should prioritize strategies that enhance teacher-related and content-related interactions, where SRL plays a more prominent mediating role (45.41% and 41.54%, respectively). For peer interaction, where direct effects dominate (77.85%), educators may need to design more structured collaborative activities that explicitly leverage GAI tools.

These recommendations align with evidence from technology-enhanced experiential learning, which indicates that digital tools can complement—but not replace—practice-oriented EI when pedagogically integrated. For instance, virtual laboratories in chemistry education have shown that technology-enhanced simulations are associated with students' practical skills and educational consciousness when carefully scaffolded alongside hands-on activities (Kolil and Achuthan, 2024). Similarly, GAI tools should be positioned as complementary resources that augment rather than substitute for face-to-face clinical reasoning, peer collaboration, and instructor feedback in nursing education.

However, the benefits of GAI must be balanced against evidence that overreliance on AI dialogue systems may affect critical cognitive capabilities, including decision-making, critical thinking, and analytical reasoning (Zhai et al., 2024). Uncritical reliance on AI-generated outputs may attenuate or reverse these benefits, underscoring the need for explicit scaffolding of critical evaluation skills in our pedagogical recommendations.

For example, before class, educators can design preview tasks or problem-solving activities based on course objectives and key/difficult points, encouraging students to use GAI tools to create text, images, audio, and other content to help them understand complex medical concepts, pathogenesis, and solve the tasks. During the process, teachers need to guide students on how to use GAI tools correctly, such as by constructing thought-provoking dialogues using the Socratic method, guiding students to find answers, and critically analyzing and using the answers provided by GAI to deepen and broaden their thinking. Educators should explicitly require students to verify, critique, and synthesize GAI outputs rather than passively accepting them, while maintaining the cognitive benefits of traditional EI and leveraging GAI's supportive functions. Additionally, problem-solving activities that require students to work with GAI could be constructively designed, where students must make significant adjustments to the generated content to complete the tasks. In class, educators should integrate GAI tools or platforms with traditional EI, such as group discussions, case analyses, and role-playing, to create a more interactive teaching environment. For student-teacher interaction, educators should actively facilitate GAI-assisted consultations and feedback sessions, as this dimension shows the strongest mediating effect through self-regulated learning. Besides, educators should encourage students to be expressive in their interactions with GAI. Post-class assignments can similarly require students to use GAI tools to complete tasks. Ultimately, these strategies may enhance EI and improve educational outcomes. However, the implementation of these approaches needs to be further designed, and their effectiveness needs to be validated in future educational reforms.

Furthermore, it is recommended that educators, while facilitating students' interaction with GAI, also promote the enhancement of students' SRL capabilities in ways that may be associated with EI. Given that the mediating effect is strongest for teacher-related (45.41%) and content-related (41.54%) interactions, educators should tailor SRL support to these specific dimensions. For instance, scaffolding students' metacognitive strategies when using GAI for content exploration or teacher consultation may yield greater benefits than generic self-regulation training. Educators should focus on key facilitators of self-regulated learning, such as student wellbeing and the adoption of teaching methods that are associated with self-regulated learning. Research indicates that teaching methods such as problem-based learning, team-based learning, and the flipped classroom can promote the enhancement of SRL capabilities, whereas traditional lecture-based methods should be reduced (Arianpoor et al., 2024). Additionally, future research can explore the other specific processes through which GAI tools enhance EI, particularly the differential processes across interaction dimensions. This can provide further insights into the optimal use of GAI tools in nursing education.

## 6 Conclusion

This study found that SGI is positively associated with the EI of nursing students and identified a statistical mediating role of SRL in this association. Nursing educators could consider implementing strategies to facilitate students' interactions with GAI, enhance their self-regulated learning, and improve EI.

To potentially utilize GAI in nursing education, we recommend educators: (1) adopt a phased integration approach, beginning with pre-class GAI-assisted content curation, progressing to in-class hybrid activities where GAI outputs are critically evaluated through peer/instructor dialogue, and concluding with post-class AI-enhanced reflection logs; (2) provide explicit scaffolding, such as worked examples demonstrating how to interrogate GAI responses for clinical accuracy; and (3) balance technological adoption with human-centered pedagogy by reserving interaction time for unmediated student-instructor contact—ensuring GAI complements rather than replaces the interpersonal interactions crucial to nursing competence. Notably, given the stronger mediating effects for student-teacher (45.41%) and student-content (41.54%) interactions compared to student-student interaction (22.15%), educators should prioritize scaffolding SRL in teacher-related and content-related contexts, while designing more direct GAI integration strategies for peer collaboration.

## Data Availability

The raw data supporting the conclusions of this article will be made available by the authors, without undue reservation.
